# Frontline Treatment of the Young, Fit Patient with CLL: A Canadian Perspective

**DOI:** 10.3390/curroncol28050326

**Published:** 2021-09-30

**Authors:** Jacqueline Costello, Matthew Kang, Versha Banerji

**Affiliations:** 1 Faculty of Medicine, Memorial University of Newfoundland, St. John’s, NL A1B 3V6, Canada; Jacqueline.Costello@easternhealth.ca; 2 Eastern Health, St. John’s, NL A1B 3V6, Canada; 3 Faculty of Health Sciences, McMaster University, Hamilton, ON L8S 4L8, Canada; kangm@mcmaster.ca; 4 Joseph Brant Hospital Oncology Clinic, Hamilton, ON L7S 0A2, Canada; 5 Departments of Internal Medicine and Biochemistry and Medical Genetics, Max Rady College of Medicine, Rady Faculty of Health Sciences, University of Manitoba, Winnipeg, MB R3E 0W2, Canada; 6 CancerCare Manitoba Research Institute, CancerCare Manitoba, Winnipeg, MB R3E 0V9, Canada

**Keywords:** chronic lymphocytic leukemia, CLL, frontline treatment, algorithm, Canadian

## Abstract

From a Canadian perspective, there has been a limited discussion on the frontline management of young, fit patients with chronic lymphocytic leukemia (CLL). The prevalence of this population ranges between 2 and 22 per 100,000 persons in Canada and varies by region. Until recently, fixed-duration fludarabine-based chemoimmunotherapy (CIT) was the primary treatment option in Canada for this patient population. The ECOG1912 trial has since demonstrated that ibrutinib and rituximab therapy are as effective as fludarabine-cyclophosphamide-rituximab (FCR) in this population. The ALLIANCE trial showed that rituximab added no incremental benefit to ibrutinib. Canadian payors and physicians adopted ibrutinib monotherapy as the CLL standard of care, even in the young, fit population, although frontline ibrutinib therapy is often reimbursed by provincial public drug plans only in patients with high-risk disease or those who are unfit to receive fludarabine. Young, fit patients with CLL and their physicians may now choose between continuous ibrutinib monotherapy and fixed-duration CIT with FCR. Factors affecting this choice include patient preference and the short- and long-term toxicity profiles of both regimens, and a risk-based algorithm is provided. As new continuous-therapy options enter the market, all treatment choices present benefits and risks that must be communicated to the patient.

## 1. Introduction

Chronic lymphocytic leukemia (CLL) is characterized by the proliferation and accumulation of small, mature B lymphocytes within the blood, bone marrow, lymph nodes, and other lymphoid tissues [1]. Canadian cancer incidence statistics from 2018 observed that 1725 Canadians (rate of 6.0/100,000 population) were newly diagnosed with CLL [2]. By extrapolation, there may have been over 2000 cases in Canada based on the 2019 population. The most recent mortality statistics for Canada are from 2017, when 611 Canadians (59% male) died from CLL [3].

These data indicate the importance of optimally managing patients with CLL. While there are published review articles on the frontline management of CLL, few have discussed the young, fit patient from a Canadian perspective [4,5]. Therefore, the goal of this manuscript is to provide treating clinicians with evidence-based and practical guidance on managing their young, fit patients with CLL.

For many years, the standard frontline treatment option for the young, fit patient with CLL was fixed-duration chemotherapy or chemoimmunotherapy (CIT) [6]. Recently, the ECOG1912 clinical trial observed that continuous therapy with the Bruton’s tyrosine kinase (BTK) inhibitor ibrutinib improved overall survival (OS) when compared to FCR [7]. The clinical importance of ECOG1912, and future continuous therapy trials, is providing young, fit patients with CLL a nonchemotherapy treatment option, especially in high-risk individuals. This change in clinical practice is made even more important in this patient population as it opens a dialogue and enables the patient to be more involved in their treatment choice.

## 2. Defining the Young, Fit Patient with CLL

For the purpose of this review, the young, fit patient with CLL is defined as a patient 65 years of age and younger; has a Cumulative Illness Rating Scale (CIRS) score of less than 6; has an Eastern Cooperative Oncology Group (ECOG) performance status of 0 to 2; and is often free of cardiac and renal disease (creatinine clearance ≥ 60 mL/min). Clinical trials have frequently used age 65 years or younger as a cutoff and have often limited participants to those with an ECOG performance status of 2 or lower [8,9]. It is acknowledged that some clinicians may employ a more general approach to assessing fitness, combining a patient’s overall appearance and ease of movement.

Risk assessments for patients with CLL can start by using the Rai or Binet staging systems—the Rai system is more commonly used in Canada [10]. Additional prognostic parameters include biomarkers (ie, IGHV mutational status, serum β2-microglobulin) and the presence of del(17p) and/or TP53 mutations [11]. Among these, IGHV status, del(17p), or TP53 mutations are considered predictive, and most Canadian clinicians use these biomarkers to guide individualized therapy. At present, IGHV mutational status, del(17p), and TP53 testing appear sufficient to guide treatment in the young, fit population. Testing for all is generally available across Canada, albeit with limited regional access and funding for TP53 testing. The CLL-international prognostic index (CLL-IPI) score is a prognostic score derived to identify high-risk disease by leveraging IGHV mutational status, age, stage, serum β2-microglobulin, and the presence of del(17p) and TP53 mutations. This cumulative score incorporates clinical, biochemical, and molecular profiling and allows prediction of 5-year and 10-year OS based on risk stratification [12]. These outcomes were largely based in an era when chemotherapy was used. Although the CLL-IPI score is not widely used in Canada, and while it cannot be applied against all treatments, it is gaining momentum as these tests become more available and may be required for entry into some clinical trials to better stratify patients. Further investigation is needed to evaluate the 5- and 10-year risk stratified predictions with novel therapies.

**Figure 1:**
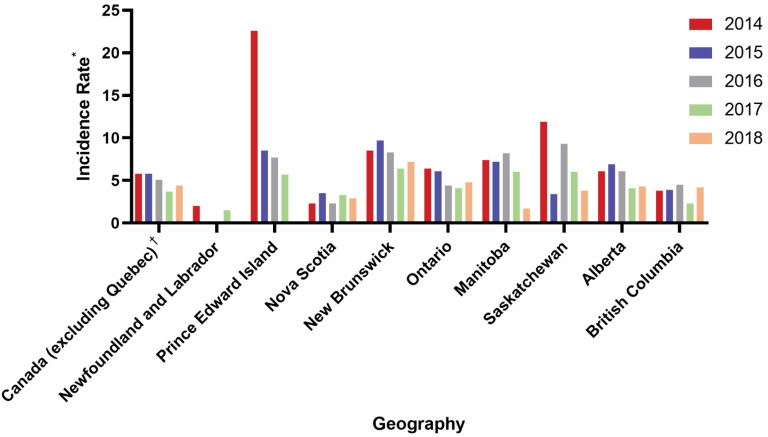
Regional CLL incidence rate of Canadians aged <65 years from 2014 to 2018. Data from [2]. (* Rate per 100,000 population: cancer incidence refers to the number of new cases of primary malignant neoplasms in a population over the given year. Cancer incidence rates for both sexes were produced using the total population estimate for both males and females; ^†^ Cancer incidence data for Quebec are not available for diagnosis years after 2010. Cancer incidence estimates for Canada excluding Quebec were produced for all diagnosis years in this graph).

Although the young, fit patient is defined above, grey zones within a clinical practice are common. Patients between the ages of 65 and 70 years may warrant being treated as young and fit, or not, depending on individual characteristics and comorbidities. In addition, a patient’s functional age may vary: for example, a 65-year-old with multiple comorbidities may have a similar health status to a healthy 80-year-old. The proportion of the 2018 Canadian population with CLL that would be considered young and fit also varies by region [13], with incidence rates ranging from between 2 and 4 per 100,000 population in Manitoba, Nova Scotia, Saskatchewan, and British Columbia, to greater than 4 to 7 per 100,000 population in Alberta, Ontario, and New Brunswick (Figure 1). These data indicate potential disparities in registries capturing the incidence of cancers, such as CLL, across the provinces and territories of Canada. Ongoing advocacy is required to ensure that cancer incidence is captured by these databases accurately.

The young, fit CLL population is associated with several advantages for treatment, including fewer comorbidities and the ability to tolerate CIT and treatment-related toxicities. However, few clinical trials have focused on this population to truly explore the optimal treatment choices in the context of their circumstances and needs, which may include employment, lifestyle, the impact of delayed adverse events, and overall patient preference.

## 3. First-Line Treatment Evolution in the Young, Fit Population

Until recently, fixed-duration fludarabine-based CIT was the primary treatment option in Canada for young, fit patients with CLL. However, over the past decade, foundational research has identified key pathways that can be targeted in patients with CLL. For example, B-cell receptor (BCR) signalling is essential for the proliferation and survival of CLL cells [6]. Kinases immediately downstream of the BCR, such as spleen tyrosine kinase (SYK) and phosphatidylinositol 3-kinase (PI3K), along with downstream amplification kinases like BTK are activated in many patients with CLL and appear essential to several CLL survival pathways [6]. In addition, CD20, a protein expressed on the surfaces of most B-cell malignancies including CLL, participates in BCR activation and signalling [14]. Chronic lymphocytic leukemia is also characterized by high levels of B-cell lymphoma 2 (BCL-2) protein, which is involved in inactivating apoptosis [14] and allowing CLL cells to evade cell death. Furthermore, loss of function of tumour suppressor genes such as TP53, ataxia telangiectasia mutated (ATM), and other somatic gene mutations (ie, SF3B-1) or complex karyotypes lead to chemotherapy-resistance in CLL [15,16]. A number of clinically approved agents have been developed to target these pathways and proteins, including acalabrutinib (BTK inhibitor), ibrutinib (BTK inhibitor), idelalisib (PI3Kδ inhibitor), obinutuzumab (anti-CD20 antibody), rituximab (anti-CD20 antibody), and venetoclax (BCL-2 inhibitor). 

**Figure 2:**
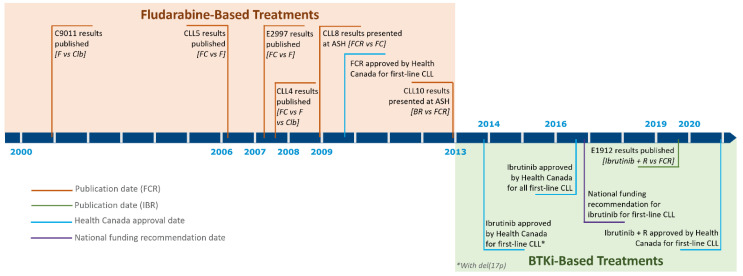
Timeline of therapeutic options for young, fit patients with CLL. BR = bendamustine and rituximab; BTKi = BTK inhibitor; Clb = chlorambucil; F = fludarabine; FC = fludarabine and cyclophosphamide; FCR = fludarabine, cyclophosphamide, and rituximab; R = rituximab.

Currently, evidence for frontline treatment in the young, fit CLL population is lacking for the majority of these agents. Most studies have involved the older, less fit population, or have been performed in the relapsed/refractory setting [17,18,19,20,21,22]. The pivotal trials that have directly impacted the frontline treatment of young, fit patients with CLL are shown in Table 1. Figure 2 illustrates the timeline of events that have influenced treatment availability for this population.

Considerable changes have occurred over the past two decades. Before the availability of novel targeted therapies, chlorambucil was the standard of care for 40 years [28]. In 2000, the CALGB 9011 trial demonstrated the superior progression-free survival (PFS) of fludarabine over chlorambucil, and fludarabine became the new standard of care, despite not improving OS [28]. Six years later, the combination of fludarabine and cyclophosphamide (FC) showed incremental PFS benefit over fludarabine monotherapy in CLL5 [24], reinforced shortly thereafter by both E2997 [25] and CLL4 [23], although, again, no differences were seen in OS.

**Figure 3:**
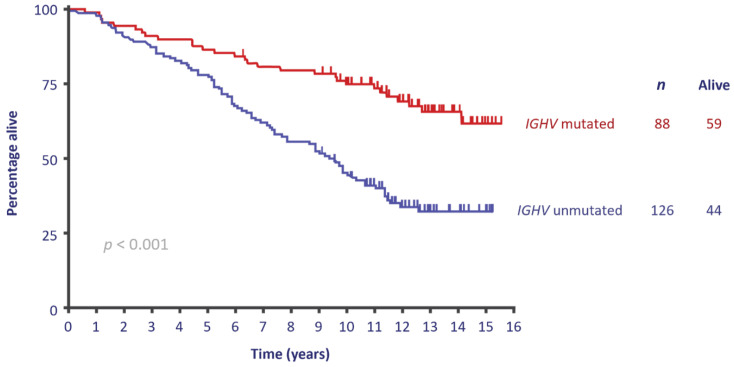
Survival with FCR treatment according to IGHV mutation status. Adapted from [30].

The addition of rituximab to FC turned chemotherapy into chemoimmunotherapy, and CLL8 was the first trial to show a survival benefit for fludarabine, cyclophosphamide, and rituximab (FCR) over FC [29]. CLL10 compared FCR to another commonly used CIT, bendamustine and rituximab (BR). FCR was found to have significantly longer PFS in the intention-to-treat (ITT) population, and especially in the younger population [8], firmly entrenching FCR as the standard of care for young, fit patients with CLL. Long-term results have further confirmed its efficacy (Figure 3).

**Figure 4:**
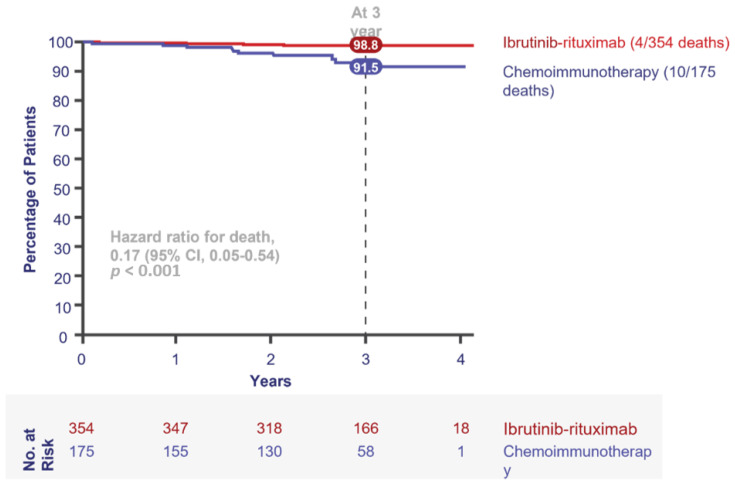
Overall survival in E1912 (intention-to-treat population). Adapted from [31]. CI = confidence interval.

**Figure 5:**
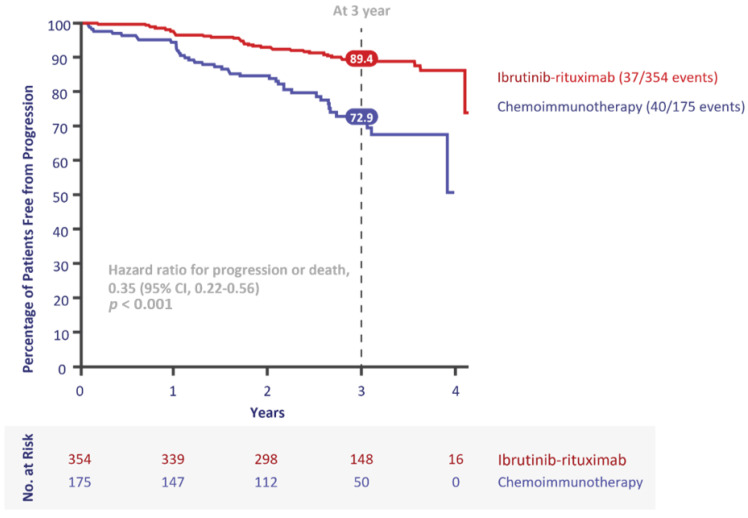
Progression-free survival of patients with CLL in E1912. Adapted from [31].

The ECOG1912 study published in 2019 comparing FCR to ibrutinib and rituximab (IR) was practice-changing [7]. For the first time, young, fit patients could be treated without chemotherapy, while benefitting from improved PFS and OS. ECOG1912 randomized treatment-naïve patients with CLL aged 70 years or younger in a 2:1 ratio to receive six cycles of IR (after a single cycle of ibrutinib alone) followed by ibrutinib alone until disease progression, or six cycles of FCR. At three years, there had been four deaths in the ibrutinib arm (one due to CLL) and 10 in the FCR arm (four due to CLL and two to therapy-related acute myeloid leukemia) (Figure 4). The statistically significant improvement in OS is a main reason Canadian clinicians readily adopted ibrutinib in their young, fit patients with CLL. There was also a significant difference in PFS seen in the IGHV-unmutated population (Figure 5), further highlighting the population where ibrutinib provides the most benefit versus FCR. There was no difference in PFS in IGHV-mutated patients, allowing clinicians and patients to engage in a dialogue on preferred treatment characteristics and to discuss the treatment goals in this population, including balancing the short- and long-term risks of time-limited chemotherapy versus continuous ibrutinib-based therapy.

During the ECOG1912 trial, therapy with ibrutinib occasionally needed to be adjusted or paused for adverse effect management, in a manner as described in the Product Monograph. Additional ECOG1912 results presented at American Society of Hematology (ASH) Annual Meeting & Exposition 2019 [31] showed that patients in whom therapy was held or discontinued for reasons other than progressive disease (after receiving a median of 15.1 months of ibrutinib therapy) obtained an additional 23 months (median) of PFS benefit without further treatment. While evidence is still being generated, this raises the question of time-limited therapy with BTK inhibitor, and whether it may be feasible to restart treatment after a prolonged treatment-free interval. The RESONATE-2 and PCYC-1102 clinical trials provide important long-term evidence for the safety and efficacy of ibrutinib in patients with CLL, including in those with high-risk features [32,33]. However, the studied populations were older and, as such, are out of scope for this review, which is focused on the young, fit patient.

The question of whether rituximab adds incremental benefit to ibrutinib alone was addressed in the ALLIANCE trial (A041202), albeit in an older but equally fit population [20]. This trial showed no difference in outcomes between ibrutinib alone versus IR. As a result, Canadian payors and physicians have adopted ibrutinib monotherapy as the CLL standard of care, even in the young, fit population. In the United States, after the E1912 results came out, the National Comprehensive Cancer Network (NCCN) guidelines altered their treatment recommendations in favour of frontline ibrutinib in young, fit patients.

## 4. Provincial Differences in Coverage

In Canada, public and private formularies, which regulate drug reimbursements, play a significant role in how novel therapeutics are used. Listing criteria in the various provincial formularies (which list drugs that are paid for by the government for eligible groups) vary widely, and their initial inclusion of a BTK inhibitor occurred over a period of four years, with the first funding of ibrutinib occurring in British Columbia in 2016, and most recently reimbursed in Prince Edward Island in 2020 [34]. For previously untreated patients with CLL, the reimbursement criteria implemented by provincial public drug plans across the country have generally limited the use of BTK inhibitors to patients who have high-risk disease features (independent of age) and/or those who are unfit to receive fludarabine (which might include age). 

At the same time as these funding criteria were being developed, Canadian physicians were gaining access to cytogenetic and molecular profiling, including IGHV mutation status, TP53 mutation status, and the fluorescence in situ hybridization (FISH) panel that detected the presence of the del(11q), del(13q), del(17p), and trisomy 12 mutations. Access to these important biomarkers also varies by province. More recently, the Canadian Agency for Drugs and Technologies in Health (CADTH) has updated its treatment algorithm to include consideration of novel therapies in younger, not fit for FCR, high-risk (such as del[17p], TP53-mutated, and IGHV-unmutated) patients with CLL.

In summary, treatment of the young, fit patient with CLL has evolved to include two primary options: fixed-duration CIT with FCR, or continuous BTK inhibitor monotherapy. Treatment choice is individualized based on cytogenetic and molecular risk profiling as well as on age, fitness, and, importantly, patient preferences.

## 5. The Canadian Landscape Today

There are many factors to consider when treating a young, fit patient with CLL. Molecular status, CIT eligibility, patient preferences, quality of life, duration of treatment, sequencing, short-term and long-term safety implications, insurance availability, and transplant eligibility are all important considerations. In addition, the young, fit population tends to be highly engaged in the treatment decision process and requires conversations with their oncologists to make decisions based on their individual situations and within provincial regulations. 

In current Canadian practice, FCR is being chosen less often than previously, even in patients with low-risk features, due to toxicity issues and the long-term risk of secondary malignancies [35]. While the risk of lifetime acute myeloid leukemia and myelodysplastic syndromes was once believed to be about 1% after FCR, retrospective studies have found that lifetime risk actually ranges between 4% and 10% [36]. Other long-term issues with FCR include the potential for severe marrow toxicity and the need for immunoglobulin support after therapy [11]. 

BTK inhibitors demonstrate a different toxicity profile compared to CIT and choosing the right treatment for each individual to maximize efficacy and minimize toxicity is a key goal. Care must be taken in deciding to use BTK inhibitors in patients with moderate to severe bleeding disorders; concomitant use of anticoagulant/antiplatelet agents; prior Grade 3 or 4 atrial fibrillation; unstable cardiovascular disease such as hypertension or new ischemic heart disease; chronic skin conditions; and compliance with oral therapy. Other considerations of continuous therapy with an oral BTK inhibitor include the risk of skin cancer; the need for patients to see their oncologists or family physician at least every three months for vital sign measurements; the importance of interrupting BTK inhibitor therapy when surgery is planned; and the necessity of education for both patients and other health care professionals. 

However, the availability of continuous oral therapies, such as ibrutinib, is an advantage when one considers patient values and nation-wide accessibility. Firstly, oral therapies provide convenience of at-home treatment to the patient with CLL and/or their caregiver [37,38]. In addition, these therapies preclude the need to travel for treatment appointments, which is a particular benefit to patients in large catchment areas that have challenges in travelling distances, and relieve the stress of frequent travel [37,39,40]. Finally, these options allow patients with CLL to continue working and not have to take time off work. This reduces disruptions to a routine schedule and helps maintain a sense of normalcy, providing a psychological benefit to many patients with CLL [37,38,39,40].

**Figure 6:**
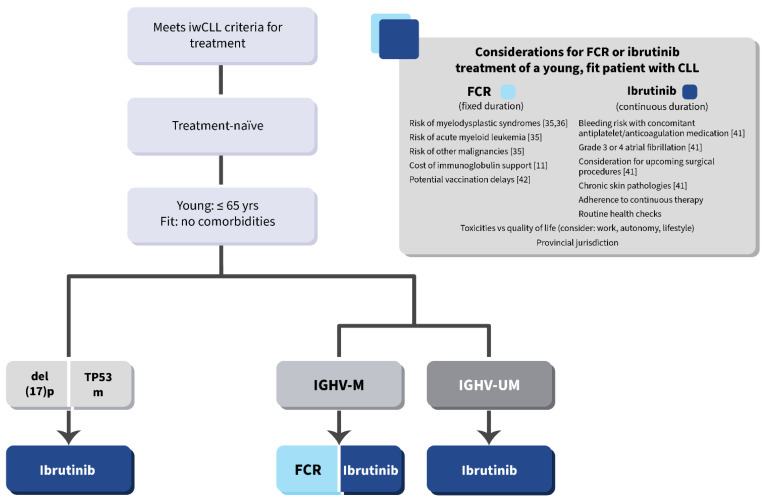
Frontline treatment algorithm for young/fit patients with CLL. iwCLL = International Workshop on Chronic Lymphocytic Leukemia.

Shared decision-making is vital for any patient but may provide relatively higher value to the young, fit CLL population. While fixed-duration chemotherapy-based therapy has the potential for long-term disease control in some low-risk patients, long-term toxicity may arise. On the other hand, continuous oral targeted therapy has the potential for long-term disease control, and this needs to be balanced with toxicities that may arise in continuous therapies [11,35,36,41,42]. Each has benefits and concerns, and each patient is different. A risk-based algorithm is provided in Figure 6, with further practical considerations outlined in the associated table.

## 6. Conclusions and Future Directions

Two decades ago, fludarabine monotherapy was associated with a 31% PFS at three years in young, fit patients with CLL [26]. Today, ibrutinib has been shown to result in an 89% PFS at three years in the same population [7]. Treatment of patients with CLL has come a long way and medicine never stops advancing. There are emerging therapies and novel combinations on the horizon, including second-generation and noncovalent BTK inhibitors permitting optimization of this important drug class [43,44,45,46].

As a clinician considers the appropriate therapy for their young, fit patient, risks and benefits need to be effectively communicated. For example, a 6-month course of FCR, while challenging to tolerate, may have the ability to achieve long-lasting remissions in certain low-risk populations without the need for retreatment [30]. Continuous oral BTKi therapy, on the other hand, may result in long term disease remission in certain patients with high-risk features [7,31,33], but, by its very nature, presents a low but real risk of mutations in the binding site which may give rise to a higher risk of treatment-resistant disease [9,22]. This risk may be less concerning in the future with the development of novel BTK inhibitors which bypass these resistant mechanisms [46]. Finally, novel combinations, such as those that include BCL-2 inhibitors, present the possibility of durable response, in some patients, with time-limited therapy [47,48,49]. 

As new agents and combinations become available, the physician and patient will both benefit through the ability to further individualize treatment. Age, fitness, comorbidities, and molecular status characterize patients with varying likelihoods of therapeutic success, and growing numbers of options increase the likelihood the most appropriate treatment for every CLL patient has been chosen based on disease characteristics, provincial jurisdiction, and, most importantly, patient-related factors and benefits.

## Figures and Tables

**Table 1:** Pivotal first-line trials in young, fit patients with CLL that affected the Canadian landscape.

Trial	Agents	ORR	Median PFS	Median OS	Del(17p) Inclusion	Median Age, Years	*n*	Median Follow-Up, Years
CLL4 [23]	FC vs. F vs. Clb	94% vs. 80% vs. 72%	36% vs. 10% vs. 10% at 5 yrs	34.2% vs. 36.6% vs. 30.0% events	7% vs. 8% vs. 6%	65 vs. 64 vs. 65	777	3.4
CLL5 [24]	FC vs. F	94% vs. 83%	48 vs. 20 months	80.3% vs. 80.7% at 3 yrs	NA	58 vs. 59	375	1.8
E2997 [25]	FC vs. F	74.3% vs. 59.5%	31.6 vs. 19.2 months	79% vs. 80% est at 2 yrs	10% vs. 9%	61 vs. 61	278	2.17
C9011 [26]	F vs. Clb	63% vs. 37% at 5.2 yrs	20 vs. 13 months	63 vs. 59 months	NA	64 vs. 62	544	~15
Knauf et al. [27]	B vs. Clb	34.6% vs. 29.9%	21.2 vs. 8.8 months	NR vs. 78.8 months	NA	63 vs. 64	319	4.5
CLL8 [9]	FCR vs. FC	90% vs. 80%	56.8 vs. 32.9 months	NR vs. 86 months	7% vs. 10%	61	817	5.9
CLL10 ≤ 65 years [8]	BR vs. FCR	98% vs. 95%	38.5 vs. 53.6 months	NA	0%	≤ 65	367	3.1
ECOG-ACRIN 1912 [7]	Ibr + R vs. FCR	95.8% vs. 81.1%	89.4% vs. 72.9% at 3 yrs	98.8% vs. 91.5% at 3 yrs	0%	56.7	529	2.8

B = bendamustine; BR = bendamustine and rituximab; Clb = chlorambucil; F = fludarabine; FC = fludarabine and cyclophosphamide; FCR = fludarabine, cyclophosphamide and rituximab; Ibr = ibrutinib; ORR = overall response rate; R = rituximab.
